# Orthologous surface proteins from *Mycoplasma hyopneumoniae *and *Mycoplasma flocculare*: *in silico *comparison and heterologous expression of differential extracellular domains

**DOI:** 10.1186/1753-6561-8-S4-P157

**Published:** 2014-10-01

**Authors:** Carolina Martello, Fernanda Leal, Veridiana Virginio, Luciano Reolon, Irene Schrank, Arnaldo Zaha, Henrique Ferreira

**Affiliations:** 1 Centro de Biotecnologia, Universidade Federal do Rio Grande do Sul, Porto Alegre, RS, Brazil

## Background

*Mycoplasma hyopneumoniae *and *Mycoplasma flocculare *are two closely related mycoplasma species often found in the porcine respiratory tract[1]. However, *M. hyopenumoniae *is pathogenic, being the causative agent of enzootic pneumonia, while *M. flocculare *is a commensal bacterium. Enzootic pneumonia is a contagious respiratory disease characterized by chronic cough, growth retardation, low mortality, and a high morbidity. It causes significative economic losses in pig industry worldwide. Some of main interactions between the host and the bacteria are mediated by surface proteins, and a comparison of surface proteins between these two mycoplasmas species can lead to identification of determinants of pathogenicity or commensalism.

## Methods

In this work the deduced amino acid sequences of *M. hyopneumoniae *and *M. flocculare *surface proteins [1,2] were aligned and comparatively analyzed. Comparative analysis was made by sequencing alignments using ClustalW algorithm available in the MEGA 5.05 software package. Orthologous pairs of interest were selected based on the differential presence of amino acid stretches ≥ 5 residues or regions of significantly reduced homology in comparison to flanking sequences. Topology analyses including predictions of extracellular and transmembrane domains were performed using the TopPred, phobius and TMHMM programs. The differential domains of the selected orthologous pairs were amplified by PCR from genomic DNA of the respective species and these amplicons were cloned into the pGEX4T-3 plasmid by *in vivo *homologous recombination in *Escherichia coli *KC8 [3]. The recombinant plasmids were expressed in *E. coli *strains adequate to heterologous expression, and the expressed recombinant polypeptides were purified by affinity chromatography and thrombin cleavage.

## Results and conclusions

A total of 170 putative surface protein sequences with identified orthologs in both *M. hyopneumoniae *and *M. flocculare *(MHP and MF, respectively) were analyzed. From this survey, three pairs of orthologs were selected for functional analysis, based on the presence of differential amino acid stretches in predicted extracellular domains. In the first pair (MHP7448_0556 and MF_00306), the *M. flocculare *protein presents an exclusive 53 aa-long stretch in its N-terminal extracellular end. In the second pair (MHP7448_0094 and MF_00500) less conserved N-terminal sequences (40.5% identical) are present, in comparison to the rest of the proteins (80% identical). And, finally, the third pair (MHP7448_0612 and MF_00357) showed an 114 aa-long stretch with 35% identity between orthologs, flanked by regions with 70%-80% identity. This third pair of orthologs had the differential domains (with 306 bp and 393 bp, respectively) cloned and expressed in *E. coli*. Star and pLysE strains. GST-tagged recombinant polypeptides of 37 kDa and 40 kDa were expressed and, after purification and cleavage with thrombin, 11.4 kDa and 14.7 kDa polypeptides were recovered for MHP7448_0612 and MF_00357, respectively. The differential domains of the other two pairs of selected orthologs will be also cloned and expressed in *E. coli *and all the recombinant polypeptides from *M. hyopneumoniae *and *M. flocculare *produced will be used for immunization of mice, in order to get evidence of potential differences between the immune responses induced by each ortholog.
